# Compressive Property of Additively-Manufactured Micro-Architectures with X-Type Lattice Unit Cell

**DOI:** 10.3390/ma15113815

**Published:** 2022-05-27

**Authors:** Yong-jing Wang, Chen-xin Feng, Zhi-jia Zhang, Dan Qian, Zhong-xiao Song

**Affiliations:** 1 State Key Laboratory for Mechanical Behavior of Materials, Xi’an Jiaotong University, Xi’an 710049, China; yongjing@xjtu.edu.cn (Y.-j.W.); zhangzhi151@mail.xjtu.edu.cn (D.Q.); 2 State Key Laboratory for Strength and Vibration of Mechanical Structures, Xi’an Jiaotong University, Xi’an 710049, China; 3120302067@stu.xjtu.edu.cn

**Keywords:** micro-architectures, porous materials, structural, additive manufacturing, mechanical properties

## Abstract

In this paper, novel micro-architectures with X-type lattice unit cell (namely, face-centered cubic (FCC), and X-type) are constructed and prepared by additive manufacturing technology. The compression behaviors of micro-architectures are explored in detail by experimental measurement and theoretical prediction. It is found that the strength of FCC micro-lattice structure is higher than that of the X-type micro-lattice structure with the same relative density. The X-type micro-lattice structure exhibits a zero Poisson’s ratio during compression deformation. In addition, the compressive strength and energy absorption efficiency of proposed micro-architectures shows a higher advantage over other previously cellular materials in a map for material selection.

## 1. Introduction

Lightweight cellular materials have received tremendous research attention in recent decades owing to their remarkable mechanical performance and multi-functional characteristics, which can inspire the development of structuring technologies for next-generation construction, automotive, aerospace, bionic structures, and medical applications [1,2,3,4,5]. Motivated by the distinctive advantages of natural materials, much effort has been devoted to creating advanced lightweight cellular structures.

Recent advancements in the technical capability for fabricating materials at multi-scale scales (from nanometer to meter) have pushed toward the development of scientifically conceived materials (also called ‘architectures’) with mechanical and multifunctionality that cannot be found in nature [6,7,8]. These multifunctionalities not only depend on the microstructure (unit cell) of the architectures under consideration, but also the physical properties of the base materials. Due to their orthotropic and three-dimensionally open-pored characteristics, architecture structures integrating structural and function features are considered the most typical and promising constructions [9].

The current literature on lattice structure is focused on their structure design [10], mechanical properties [11,12,13], fabrication technologies [14], etc. For structure design, tetrahedral [15], pyramidal [16], octet lattice [17], 3D-Kagome [18], metal textile [19], cuttlebone-like lattice [20], and open-cell foam [21] are the main topologies used to construct the lattice truss structures. Nevertheless, it is still unsatisfactory that the critical buckling loads of lattice structures, categorized as either truss- or plate-based, are sensitive to manufacturing defects [22,23]. The energy absorption performances of these lattice structures have yet to be improved. Under quasi-static compression, for example, a pyramidal lattice is deformed by the stretching of the lattice strut and collapse by buckling (Euler or plastic), with the stress rapidly reducing once the peak stress is reached. The strut members in pyramidal configurations are independent to a great extent. When the relative density of the core is small, the lattice strut is easy to rotate. Hence, it is of great significance to explore lattice structures with a high bearing capacity and low defect sensitivity [24,25]. The two-dimensional constrained nodes introduced into the strut elements of novel lattice configurations, called X-type, result in a change in the degree of freedom of the struts. The results show that the plastic deformation stability of an X-type lattice under compression and shear are better than that of a pyramid lattice [26,27]. Up to now, however, little attention has been paid to using X-type lattice unit cells to construct architecture structures.

In this work, micro-architectures with X-type lattice unit cells (namely, face-centered cubic (FCC), and X-type) are designed and made with 3D printing technology. The compression behaviors of micro-architectures are investigated by experimental measurement and theoretical prediction.

## 2. Fabrication and Experiments

### 2.1. Structure Description

**Figure 1:**
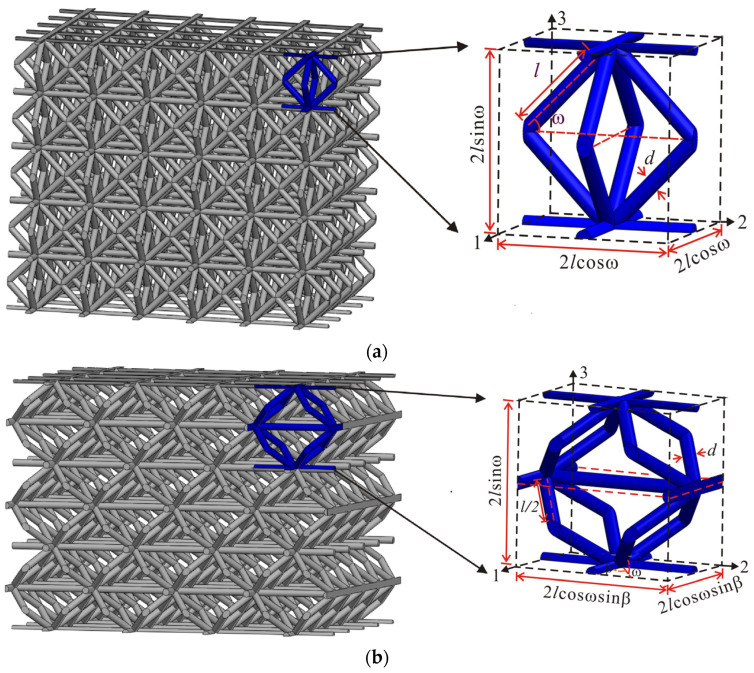
Geometric diagram and actual image of two designed architecture materials: (**a**) the unit cell of FCC lattice; (**b**) the unit cell of X-type lattice.

In this work, two typical unit cells, i.e., X-type lattice and face-centered cubic (FCC) are proposed as shown in Figure 1. The X-type lattice unit cell can be obtained by rotating the FCC unit cell 45 degrees around axis 1 and then 90 degrees around axis 2. The geometrical configurations and parameters of unit cells are shown in Figure 1a,b. The micro-architectures with consistent the outline dimensions of *W* × *L* × *H*, the truss length *l*, the angle of inclination β for FCC lattice and β and ω for X-type lattice as well as various truss diameter *d*. All samples are listed in Table 1.

### 2.2. Preparation Process and Materials

**Figure 2:**
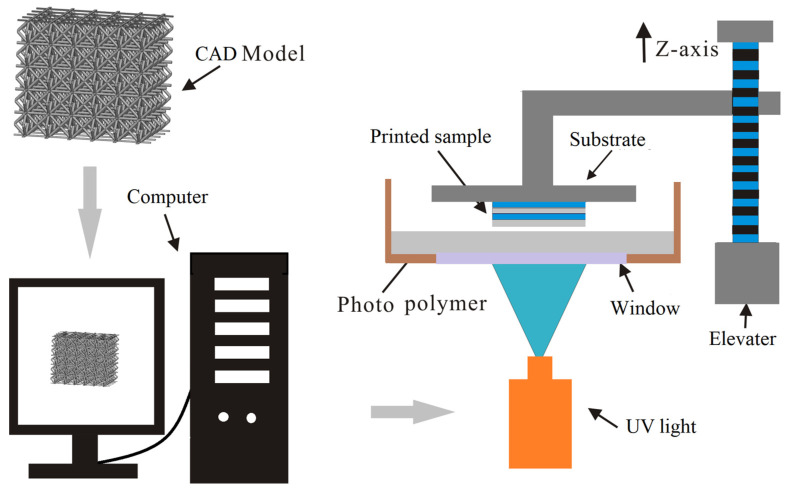
Schematic of a typical additive manufacturing process using an Autodesk Ember 3D printer.

**Figure 3:**
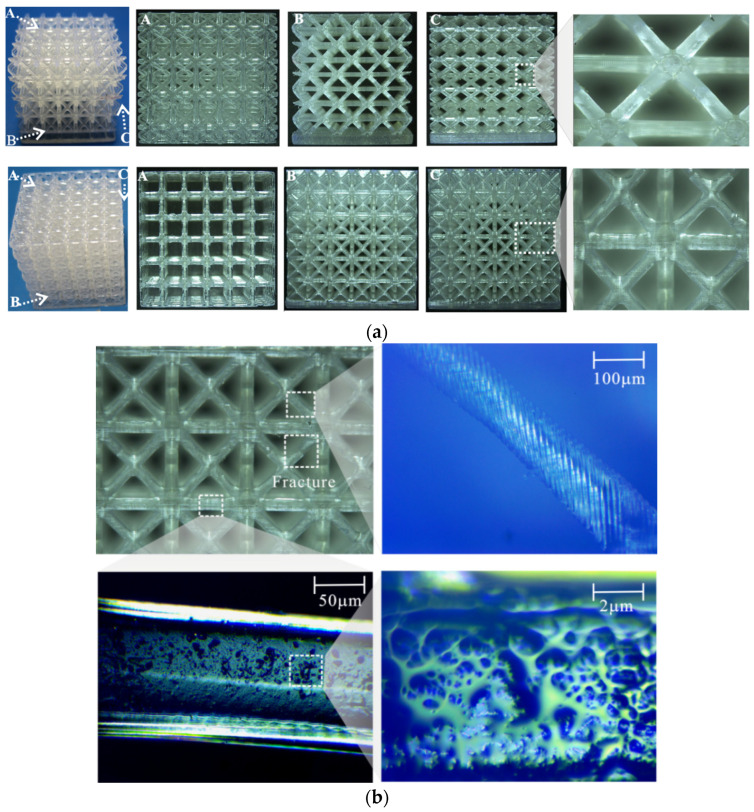
(**a**) Specimen A–C fabricated through 3D printing; (**b**) SEM of micro-architectures.

The proposed micro-architectures were prepared by using a AutodeskEmber (desktop DLP) 3D printer (Autodesk Inc., San Rafael, CA, USA) as shown in Figure 2. Photopolymer resin (PR) 48 was chosen for this study. The key process parameters are as follows: an LED projector (Autodesk Inc., San Rafael, CA, USA) with 405 nm wavelength and 5 W power, and a printing layer thickness of 20 μm. All samples were washed with solvent and dried with compressed air. Subsequently, all the samples were solvent-cleaned (isopropyl alcohol, IPA) and post-cured with ultraviolet light for 10 min to enhance the mechanical properties of the samples. To understand the mesostructure of the as-prepared samples, scanning electron microscopy (SEM) was performed using a Quanta FEG250 microscope (FEI Inc., Hillsboro, OR, USA). Figure 3a illustrates the as-built micro-architectures with high geometric accuracy. The microstructures of the as-built micro-architectures were characterized by SEM as shown in Figure 3b. The uneven diameter of the struts can still be observed. It may result from the creep induced by their own self-weight in the viscous fluid during the complex solidification process. At the same time, due to the continuous layer-by-layer manufacturing process, there are ripples on the struts surface. These defects would affect the mechanical properties of the desired structure.

### 2.3. Experimental Test

The quasi-static compressive tests were performed on the prepared specimens with a hydraulic testing machine (Zwick/Roell, 2.5 KN, Ulm, Germany) at room temperature. A compressive load was applied at a rate of 0.5 mm/min to ensure the implementation of quasi-static compression with a nominal strain rate of 10^−3^ s^−1^, according to the guidelines of ASTM STP C365 [28]. The compressive load and compressive displacement were obtained using a computer acquisition system with 10 data points per second. In order to obtain the deformation history of the specimens, the compressive process of each specimen was recorded by a video camera that was placed horizontally in front of the test specimen. The tests were repeated three times, and the results were averaged with at least 70% of the compressive strain. The constituents material of the PR48 employed in simulation are shown in Table 2 (which are provided by Colorado Photopolymer Solutions, LLC, Boulder, CO, USA).

## 3. Theoretical Model

The geometric parameters of unit cell are shown in Figure 1, and the corresponding relative density of micro-architectures can be written as

(1) $${\bar{\rho }}_{FCC}=\frac{\pi (2+\cos\omega )}{{\bar{l}}^{2}\cos\omega \sin2\omega }$$

(2) $${\bar{\rho }}_{X}=\frac{2\pi (2+\cos\omega (\sin\beta +\cos\beta )}{{\bar{l}}^{2}\sin2\omega \cos\omega \sin2\beta }$$

**Figure 4:**
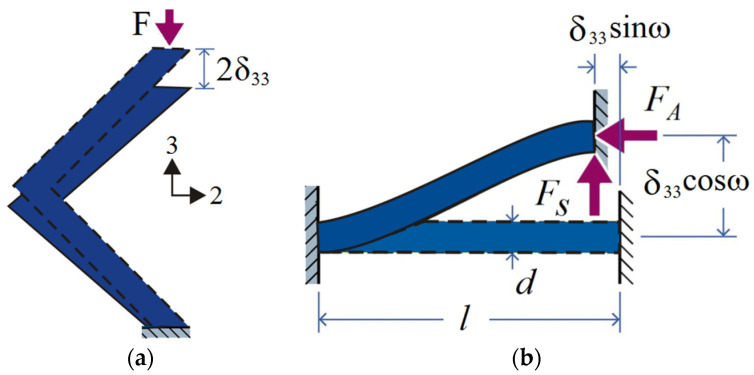
Schematic showing (**a**) the deflection of core upon application of a uniaxial compressive load; (**b**) the free-body diagram of a strut (half unit in (**a**)) subjected to compression and shear.

Consider a quarter of architecture lattice, as shown in Figure 4, with an axial compressive force *F* applied in the through thickness 3-direction. The resulting displacement associated with the application of this force is 2$\delta_{33}$. A free-body diagram for a rigid-jointed angled strut is shown in Figure 4b, in which the axial, $F_{A}$, and shear, $F_{S}$, forces occur in each of the struts. For an imposed compression displacement, the axial and shear forces in each of the angled struts are given using beam theory

(3) $$F_{A}=\frac{E_{s}\pi d^{2}\delta \sin\omega }{4l}$$

and

(4) $$F_{s}=\frac{12E_{s}I\delta \cos\omega }{l^{3}}$$

where I and $E_{s}$ are the second moment of area of the inclined strut and Young’s modulus of the material, respectively.

The total applied compression force on the unit cell of architecture lattice follows as

(5) $$\begin{array}{l} F=4(F_{A}\sin\omega +F_{S}\cos\omega )\\ =\frac{E_{S}\pi d^{2}\delta_{33}}{l}(\sin^{2}\omega +3{\left(\frac{r}{l}\right)}^{2}) \end{array}$$

where *r* is the radius of lattice strut.

The applied through-thickness stress $\sigma_{33}$ and strain $\varepsilon_{33}$ of the unit cell are then correlated as

(6) $$\sigma_{33}=\frac{F}{{\left(2l\cos\omega \right)}^{2}}$$

and

(7) $$\varepsilon_{33}=\frac{\delta_{33}}{l\sin\omega }$$

The initial modulus for the unit cell of architecture lattice $E_{33}=\sigma_{33}/\varepsilon_{33}$ follows from Equations (6) and (7) as

(8) $$E_{33}=\left\{ \begin{array}{l} \frac{E_{s}\pi \sin\omega }{{\bar{l}}^{2}\cos^{2}\omega }(\sin^{2}\omega +\frac{3\cos^{2}\omega }{{\bar{l}}^{2}}),\mathrm{BCC}\;\mathrm{uint}\;\mathrm{cell}\\ \frac{E_{S}\pi \sin\omega }{{\bar{l}}^{2}\cos^{2}\omega \cos\beta \sin\beta }(\sin^{2}\omega +\frac{12\cos^{2}\omega }{{\bar{l}}^{2}}),X\text{-}\mathrm{type}\;\mathrm{uint}\;\mathrm{cell} \end{array}\right.$$

where the non-dimensional length $\bar{l}=l/r$. When $\bar{l}=l/r$ of the member is large, the second item can be ignored. The initial modulus for the FCC depends on the angle of inclination ω, however, and the initial modulus for the FCC depends on the angle of inclination β and ω.

Equation (5) gives the compressive stress $\sigma_{33}$ applied to the unit cell in the 3-direction. The peak stress $\sigma_{33}$ of unit cell is attained when trusses yield or buckle simultaneously and can be obtained as

(9) $$\sigma_{33}=\left\{ \begin{array}{l} \frac{\pi \sin\omega ({\bar{l}}^{2}+3\cot^{2}\omega )\sigma_{c}}{{\bar{l}}^{2}{\left(\bar{l}\cos\omega \right)}^{2}},\mathrm{BCC}\;\mathrm{uint}\;\mathrm{cell}\\ \frac{2\pi \sin\omega }{{\bar{l}}^{4}\cos^{2}\omega \cos\beta \sin\beta }({\bar{l}}^{2}+12\cot^{2}\omega )\sigma_{c},X\text{-}\mathrm{type}\;\mathrm{uint}\;\mathrm{cell} \end{array}\right.$$

This compressive stress is set by either elastic buckling or yield of the struts, and may be obtained as

(10) $$\sigma_{E}=\left\{ \begin{array}{l} \frac{k^{2}\pi^{2}E_{s}I}{A_{s}l^{2}},\mathrm{bulckling}\\ \sigma_{y},\mathrm{yield} \end{array}\right.$$

where I is the second moment of area of the strut, $A_{s}$ is the strut cross-sectional area ($A_{s}=\pi d^{2}/4$). k relies on the strut end conditions (k = 2 for fixed truss or 1 for simple support truss). 

## 4. Results and Discussion

### 4.1. Compressive Responses

**Figure 5:**
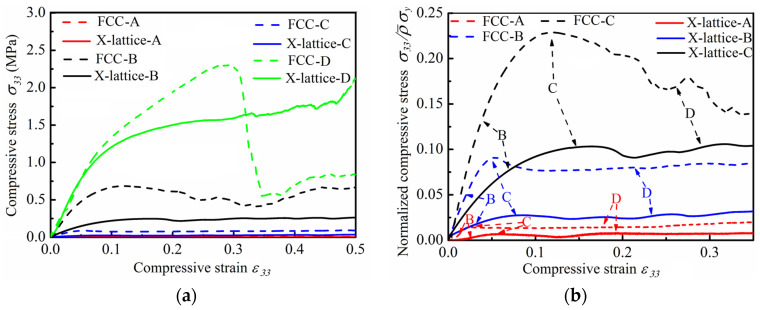
(**a**) Stress–strain curves of micro-architectures with variable relative density; (**b**) the normalized stress–strain curves of micro-architectures.

The measured compressive stress–strain responses for micro-architectures with FCC lattice unit cell and X-type lattice unit cell are shown in Figure 5a. Under continuous loading, the curves exhibit three distinct regions: initial elastic stage, inelastic stage, and stress platform stage (except for specimen D, where the brittle fracture of the specimen occurred). When the compressive strain is less than 0.05, the lattice struts are in the elastic deformation region. As the compressive strain increases further, the stress–strain curve of the micro-architectures slowly transits from the elastic region to the platform region, and then the platform region maintains itself in the range of strain 0.1 to 0.5. In addition, the critical strain of the densification zone decreases with the increase in the slender ratio of the lattice struts. The stress of FCC lattice has a certain degree of decrease after reaching the peak value. However, the X-type lattice does not appear this phenomenon, which is similar to that of foam material. Clearly, the FCC lattice micro-architectures outperform the X-type ones, which are revealed in the normalized stress–strain curve as displayed in Figure 5b.

**Figure 6:**
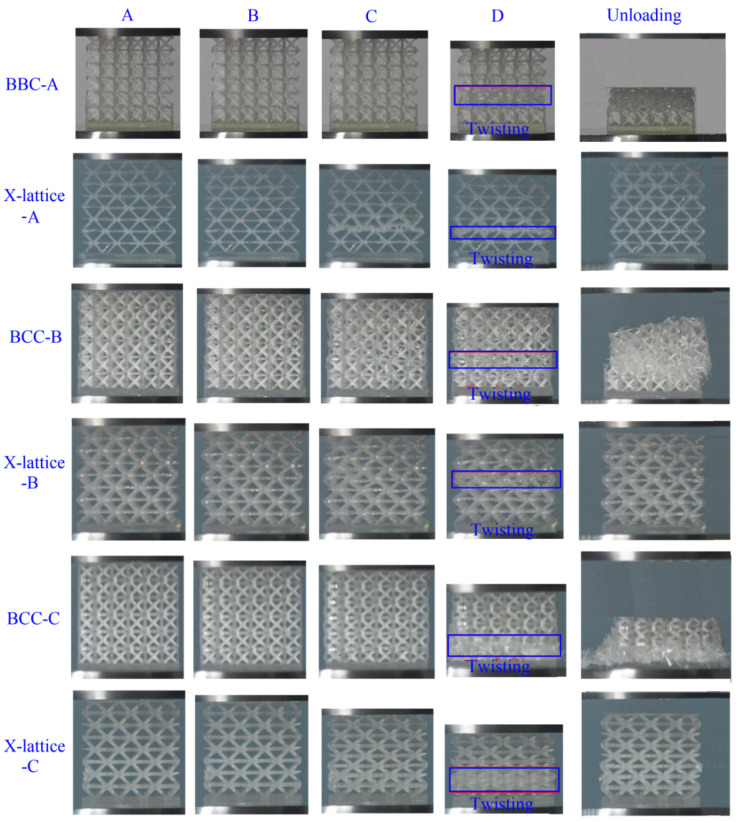
The typical deformation of micro-architectures (Sample A–D).

Figure 6 displays the deformation history of two kinds of micro-architectures with different slenderness ratios during compression. The results show that Euler buckling occurs first when the slenderness ratio of lattice truss is small. For FCC lattice, as the slenderness ratio of lattice truss increases, the brittle fracture failure first occurs in the end of sample, and gradually extends to the other end, accompanied by obvious lateral expansion. For X-type lattice, during the initial failure stage, the lattice truss near the bottom end buckles firstly. With the increase in compressive strain, the buckling lattice trusses undergo serious deformation. For example, some lattice trusses are severely distorted and are even in contact with other buckled lattice trusses. In the whole compression process, severe buckling propagates from the bottom end to the top end like waves, resulting in the gradual densification of micro-architectures. The elastic buckling allows extensive rotation/deformation around thin lattice trusses without introducing plastic strain, resulting in almost complete recovery even after compressive strain exceeds 70%. Unlike BBC lattice, with the increase in compressive strain, there is no obvious transverse expansion of the X-type lattice, the Poisson’s ratio of the X-type lattice is almost zero, and there is no fracture during the whole compression process. When the compression strain reaches 70% and the compression load is unloaded and the X-type lattice can recover to more than 90% of the original structure height, which shows that this type of micro-architecture has good compressibility and has great application value in vibration damping components.

**Figure 7:**
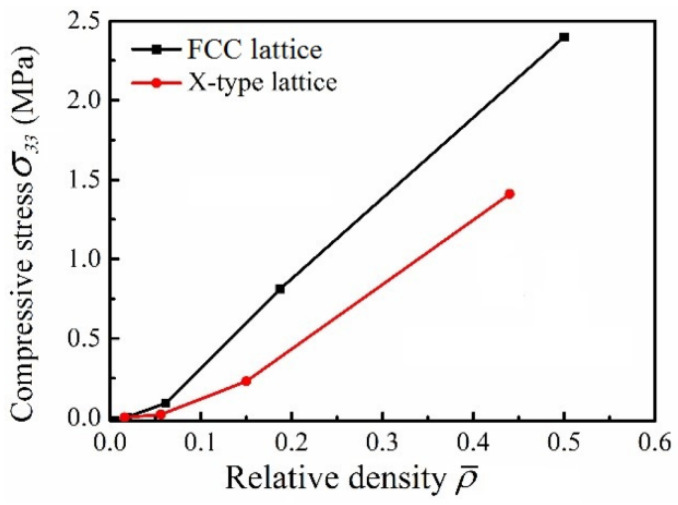
The variation in peak stress of structure with relative density.

Figure 7 shows the variation trend of the peak stress of micro-architectures with the relative density. It can be seen, with the increase in the relative density, the peak strength of micro-architectures increases. The relative density exceeds 0.15, the peak strength increases approximately linearly. At the same time, it can be found that in the low-density region, the peak strength of the two micro-architectures has little difference under the same density, but with the increase in relative density, the strength value of the FCC lattice is significantly higher than that of X-type lattice. When the relative density is 0.45, the strength value of FCC lattice is 1.5 times that of X-type lattice.

### 4.2. Failure Mode

**Figure 8:**
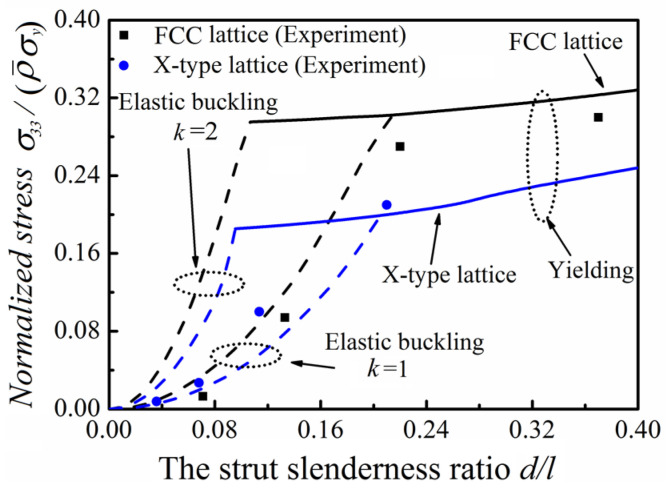
The analytical predictions of normalized failure stresses with experimental measurements.

The function relationship between the relative strength and the slenderness ratio of FCC lattice and X-type lattice predicted theoretically is shown in Figure 8. It is found that when the slenderness ratio of the strut is small, the failure mode of micro-architectures is dominated by Euler buckling, and the end constraint is closer to the simply supported constraint. The potential reason is that samples obtained by 3D printing have certain manufacturing defects. With the increase in slenderness ratio, the failure mode transition from Euler buckling to material yield and the end constraint is closer to the fixed supported constraint. Combined with the results in Table 3 and Figure 8, it can be found that the theoretical prediction is reliable.

### 4.3. Comparison with Competitive Cellular Materials

**Figure 9:**
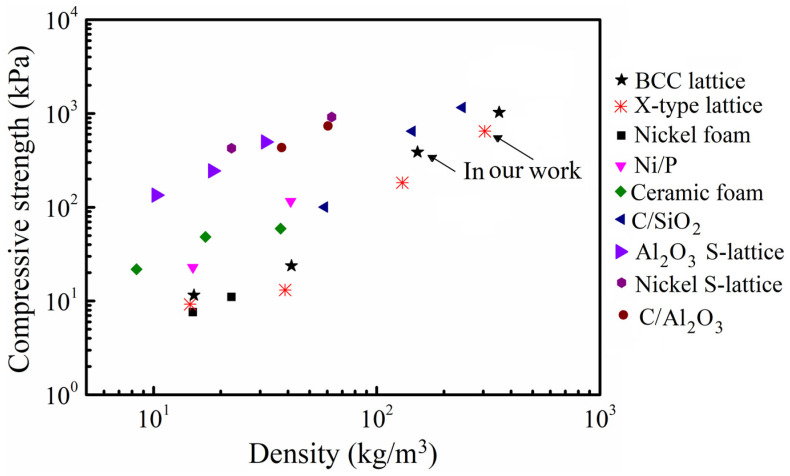
The strength versus density for micro-architectures compared with competing core topologies.

Figure 9 gives the compressive strength versus density in a map for material selection in the current literature [29]. Compared to simple nickel foam, composite foam and composite lattice materials, the as-designed micro-architectures demonstrate a higher strength. The strength of as-designed polymer micro-architectures in this work can nearly match that of nickel foams and composite foam materials in the density range of 10–500 kg/m^3^. The superior property in the micro-architectures is mainly due to the coupling of polymer and the X-type unit cell, which alter the strut end conditions and suppress strut buckling during large compressive deformation.

Energy can be dissipated by elastic-plastic buckling and fracture of lattice trusses, so it is an index of energy absorption efficiency. The energy can be dissipated through elastic-plastic buckling and fracture at the nodes, which therefore gives an indicator of the high-efficient energy absorption. In order to evaluate the energy absorption performance of two micro-architectures, the absorbed energy efficiency (AEE), η, can be quantitatively given as $\eta =\int_{0}^{\varepsilon_{a}}\sigma (\varepsilon )d\varepsilon /\sigma_{p}\varepsilon_{a}$, where σ is compressive stress, $\sigma_{p}$ is the peak stress, and *ε* is compressive strain (up to = 0.3 in this work).

**Figure 10:**
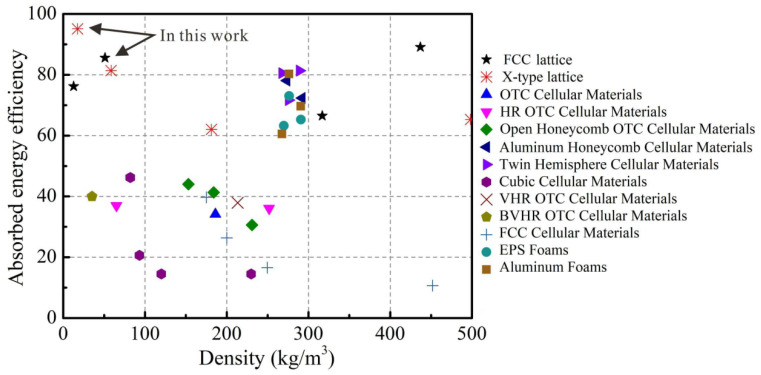
The energy absorption efficiency versus density for micro-architectures compared with competing core topologies.

In order to compare the energy absorption capability of micro-architectures, Figure 10 compares the energy absorption efficiency of different cellular material in the current literature [30,31,32,33,34], including honeycombs with different materials, octet lattices, and foam material. It is found that the polymer FCC and X-type micro-architectures have a high energy absorption coefficient in different density regions, which shows excellent energy absorption performance.

## 5. Conclusions

The FCC lattice and X-type lattice micro-architectures were proposed and prepared by 3D printing technology while the failure mode and strength were studied by theoretical analysis and experimental test methods. It was found that the strength of FCC lattice is higher than that of X-type lattice under the same relative density, and the X-type lattice exhibits zero Poisson’s ratio during compression deformation. At low relative density, Euler buckling occurs in both of the two kinds of micro-architectures. By comparing the porous materials with different topological structures, it was found that the compressive strength and energy absorption efficiency of polymer X-type lattice and FCC lattice micro-architectures have higher advantages in a map for material selection.

## Figures and Tables

**Table 1:** Configuration parameters of different lattice structures (mm).

Sample	*W*	*L*	*H*	*d*	*l*	*β^o^*	*ω^o^*
FCC-A	17.6	17.6	17.6	0.16	2.2	35.2	30
FCC-B	17.6	17.6	17.6	0.3	2.2	35.2	30
FCC-C	17.6	17.6	17.6	0.5	2.2	35.2	30
FCC-D	17.6	17.6	17.6	0.84	2.2	35.2	30
X-lattice-A	17.6	18	17.6	0.16	4.4	45	-
X-lattice-B	17.6	18	17.6	0.3	4.4	45	-
X-lattice-C	17.6	18	17.6	0.5	4.4	45	-
X-lattice-D	17.6	18	17.6	0.84	4.4	45	-

**Table 2:** Mechanical properties of PR48.

Specimens	Fresh Print	Post-Cured
Young’s modulus (MPa)	630	1380
Yield stress (MPa)	16	28
Elongation (%)	5	3

**Table 3:** Comparison of measured and predicted mechanical properties of micro-architectures.

	FCC Lattice				X-Type Lattice			
	A	B	C	D	A	B	C	D
d/l	0.07	0.13	0.22	0.37	0.04	0.07	0.11	0.19
Relative density	0.02	0.06	0.19	0.50	0.016	0.056	0.15	0.44
Theoretical prediction stress	0.002	0.130	0.89	2.70	0.0 01	0.021	0.23	1.41
Experimental test Stress	0.004	0.091	0.81	2.40	0.0 02	0.025	0.24	1.51
Experimental value error	±0.0004	±0.005	±0.03	±0.2	±0.0003	±0.004	±0.02	±0.1

## Data Availability

Not applicable.

## References

[1] Ashby M.F., Cebon D. (2007). Materials: Engineering, Science, Processing and Design.

[2] Zhang Q.C., Yang X.H., Li P., Huang G.Y., Feng S.S., Shen C., Zhang X., Jin F., Xu F., Lu T.J. (2015). Bioinspired engineering of honeycomb structure-Using nature to inspire human innovation. Prog. Mater. Sci..

[3] Ha N.S., Lu G.X. (2020). A review of recent research on bio-inspired structures and materials for energy absorption applications. Compos. Part B Eng..

[4] Ha N.S., Pham T.M., Tran T.T., Hao H., Lu G.X. (2022). Mechanical properties and energy absorption of bio-inspired hierarchical circular honeycomb. Compos. Part B Eng..

[5] Yan H.B., Zhang Q.C., Chen W., Xie G.G., Dang J.J., Lu T.J. (2020). An X-lattice cored rectangular honeycomb with enhanced convective heat transfer performance. Appl. Therm. Eng..

[6] Qi D., Hu W., Xin K., Zeng Q., Xi L., Tao R., Wu W.W. (2020). In-situ synchrotron X-ray tomography investigation of micro lattice manufactured with the projection micro-stereolithography (PuSL) 3D printing technique: Defects characterization and in-situ shear test. Compos. Struct..

[7] Yap X.Y., Seetoh I., Goh W.L., Ye P., Zhao Y., Du Z., Gan C.L. (2021). Mechanical properties and failure behaviour of architected alumina microlattices fabricated by stereolithography 3D printing. Int. J. Mech. Sci..

[8] Lee J.H., Singer J.P., Thomas E.L. (2012). Micro-/nanostructured mechanical metamaterials. Adv. Mater..

[9] Jin X., Li Y., Yan H.B., Xie X.X. (2019). Comparative study of flow structures and heat transfer enhancement in a metallic lattice fabricated by metal sheet folding: Effects of punching location shift. Int. J. Heat Mass Transf..

[10] Dong L. (2021). Mechanical responses of Ti-6Al-4V truss lattices having a combined simple-cubic and body-centered-cubic (SC-BCC) topology. Aerosp. Sci. Technol..

[11] Surjadi J.U., Gao L., Du H., Li X., Xiong X., Fang N.X., Lu Y. (2019). Mechanical metamaterials and their engineering applications. Adv. Eng. Mater..

[12] Grummon D.S., Shaw J.A., Foltz J. (2006). Fabrication of cellular shape memory alloy materials by reactive eutectic brazing using niobium. Mater. Sci. Eng. A.

[13] Wadley H.N.G., Fleck N.A., Evans A.G. (2003). Fabrication and structural performance of periodic cellular metal sandwich structures. Compos. Sci. Technol..

[14] Xiong J., Ma L., Wu L.Z., Wang B., Ashkan V. (2010). Fabrication and crushing behavior of low density carbon fiber composite pyramidal truss structures. Compos. Struct..

[15] Wallach J.C., Gibson L.J. (2001). Mechanical behavior of a three-dimensional truss material. Int. J. Solids Struct..

[16] Wallach J.C., Gibson L.J. (2001). Defect sensitivity of a 3D truss material. Scr. Mater..

[17] Dong L., Zhang S.K., Yu K.H. (2021). Ti-6Al-4V truss lattices with a composite topology of double-simple-cubic and body-centered-cubic. Eur. J. Mech. A Solids.

[18] Lee Y.H., Lee B.K., Jeon I., Kang K.J. (2007). Wire-woven bulk Kagome truss cores. Acta Mater..

[19] Zok F.W., Rathbun H.J., Wei Z., Evans A.G. (2003). Design of metallic textile core sandwich panels. Int. J. Solids Struct..

[20] Takano N., Takizawa H., Wen P., Odaka K., Matsunaga S., Abe S. (2017). Stochastic prediction of apparent compressive stiffness of selective laser sintered lattice structure with geometrical imperfection and uncertainty in material property. Int. J. Mech. Sci..

[21] Belardi V.G., Fanelli P., Trupiano S., Vivio F. (2021). Multiscale analysis and mechanical characterization of open-cell foams by simplified FE modeling. Eur. J. Mech. A Solids.

[22] Yang C., Li Q.M., Wang Y. (2021). Compressive properties of cuttlebone-like lattice (CLL) materials with functionally graded density. Eur. J. Mech. A Solids.

[23] Han B., Zhang Z.J., Zhang Q.C., Zhang Q., Lu T.J., Lu B.H. (2017). Recent advances in hybrid lattice-cored sandwiches for enhanced multifunctional performance. Extrem. Mech. Lett..

[24] Queheillalt D.T., Murty Y., Wadley H.N.G. (2008). Mechanical properties of an extruded pyramidal lattice truss sandwich structure. Scr. Mater..

[25] Queheillalt D.T., Wadley H.N.G. (2005). Pyramidal lattice truss structures with hollow trusses. Mater. Sci. Eng. A.

[26] Zhang Q.C., Han Y.J., Chen C.Q., Lu T.J. (2009). Ultralight X-type lattice sandwich structure (I): Concept, fabrication and experimental characterization. Sci. China Ser. E Technol. Sci..

[27] Zhang Q.C., Chen A.P., Chen C.Q., Lu T.J. (2009). Ultralight X-type lattice sandwich structure (II): Micromechanics modeling and finite element analysis. Sci. China Ser. E Technol. Sci..

[28] (2003). Standard Test Method for Flatwise Compressive Properties of Sandwich Cores. Annual Book of ASTM Standards.

[29] Deng B., Xu R., Zhao K., Lu Y., Ganguli S., Cheng G.J. (2018). Composite bending-dominated hollow nanolattices: A stiff, cyclable mechanical metamaterial. Mater. Today.

[30] Song J., Zhou W., Wang Y., Fan R., Wang Y., Chen J., Lu Y., Li L.X. (2019). Octet-truss cellular materials for improved mechanical properties and specific energy absorption. Mater. Des..

[31] Mieszala M., Hasegawa M., Guillonneau G., Bauer J., Raghavan R., Frantz C., Kraft O., Mischler S., Michler J., Philippe L. (2017). Micromechanics of amorphous metal/polymer hybrid structures with 3D cellular architectures: Size effects, buckling behavior, and energy absorption capability. Small.

[32] Liu Y., Schaedler T.A., Chen X. (2014). Dynamic energy absorption characteristics of hollow microlattice structures. Mech. Mater..

[33] De Sousa R.A., Gonçalves D., Coelho R., Teixeira-Dias F. (2011). Assessing the effectiveness of a natural cellular material used as safety padding material in motorcycle helmets. Simulation.

[34] Caserta G.D., Iannucci L., Galvanetto U. (2011). Shock absorption performance of a motorbike helmet with honeycomb reinforced liner. Compos. Struct..

